# Characterization of Tannic Acid-Coated AZ31 Mg Alloy for Biomedical Application and Comparison with AZ91

**DOI:** 10.3390/ma17020343

**Published:** 2024-01-10

**Authors:** Jacopo Barberi, Muhammad Saqib, Anna Dmitruk, Jörg Opitz, Krzysztof Naplocha, Natalia Beshchasna, Silvia Spriano, Sara Ferraris

**Affiliations:** 1 Department of Applied Science and Technology, Politecnico di Torino, 10129 Turin, Italy; silvia.spriano@polito.it; 2 Centro Interdipartimentale Polito BioMEDLab, Politecnico di Torino, Via Piercarlo Boggio 59, 10138 Torino, Italy; 3 Fraunhofer Institute for Ceramic Technologies and Systems IKTS, 01109 Dresden, Germany; muhammad.saqib@ikts.fraunhofer.de (M.S.); joerg.opitz@ikts.fraunhofer.de (J.O.); natalia.beshchasna@ikts.fraunhofer.de (N.B.); 4 Department of Lightweight Elements Engineering, Foundry and Automation, Faculty of Mechanical Engineering, Wroclaw University of Science and Technology, 50-370 Wroclaw, Poland; anna.dmitruk@pwr.edu.pl (A.D.); krzysztof.naplocha@pwr.edu.pl (K.N.)

**Keywords:** magnesium alloys, polyphenols, controlled degradation rate, coating, corrosion, biomaterials

## Abstract

Magnesium alloys are promising materials for bioresorbable implants that will improve patient life and reduce healthcare costs. However, their clinical use is prevented by the rapid degradation and corrosion of magnesium, which leads to a fast loss of mechanical strength and the formation of by-products that can trigger tissue inflammation. Here, a tannic acid coating is proposed to control the degradation of AZ31 and AZ91 alloys, starting from a previous study by the authors on AZ91. The coatings on the two materials were characterized both by the chemical (EDS, FTIR, XPS) and the morphological (SEM, confocal profilometry) point of view. Static degradation tests in PBS and electrochemical measurements in different solutions showed that the protective performances of the tannic acid coatings are strongly affected by the presence of cracks. The presence of fractures in the protective layer generates galvanic couples between the coating scales and the metal, worsening the corrosion resistance. Although degradation control was not achieved, useful insights on the degradation mechanisms of coated Mg surfaces were obtained, as well as key points for future studies: it resulted that the absence of cracks in protective coatings is of uttermost importance for novel biodegradable implants with proper degradation kinetics.

## 1. Introduction

Magnesium and its alloys are of interest in several biomedical applications due to their biocompatibility and biodegradability. In orthopedic applications, the elastic modulus of magnesium, which is around 40 GPa and close to the one of bone [1], can significantly reduce the stress shielding phenomenon compared to traditional implant metals. Moreover, biodegradability allows the avoidance of a second surgery for implant removal in all temporary applications such as trauma treatment, with consequent advantages for both patients and the healthcare system [2]. In cardiovascular applications, bioresorbable materials can reduce the risks associated with late thrombosis, mechanical failure, and permanent anti-platelet therapies connected with permanent implants. Moreover, degradable metals, such as magnesium and its alloys, present superior mechanical properties and biocompatibility compared to degradable polymers [3].

The main obstacle to the diffusion of biodegradable magnesium-based implants is related to too rapid and uncontrolled degradation. Moreover, the degradation of magnesium-based material is accompanied by hydrogen development and the production of hydroxide ions and reactive intermediate species (RIS), such as reactive oxygen species (ROS), reactive nitrogen species (RNS), reactive carbonyl species (RCS), and reactive sulfur species (RSS) [4,5]. These aspects, together with a significant pH increase in the degradation medium, can hamper the biocompatibility of magnesium alloys and restrict their application.

In order to control the degradation rate of magnesium and its alloys, numerous kinds of coatings have been investigated: calcium phosphates, oxides and hydroxides, metals, polymers, or composites have been considered and obtained by different techniques such as microarc oxidation (MAO), plasma electrolytic oxidation (PEO), electrodeposition, physical vapor deposition (PVD), layer by layer, sol–gel or chemical conversion, to cite some examples [6,7].

Among the possible substances to be employed for corrosion protection/control of metallic substrates, polyphenols constitute an interesting option due to their ability to bind to metals and to confer, in addition to corrosion protection, some specific biological properties such as antioxidant, anti-inflammatory, vasculo-protective and bone stimulating properties [8,9,10].

Some research works recently explored the possibility of exploiting these properties of polyphenols to obtain coatings on magnesium substrates to control their degradation rate [11,12,13,14,15,16,17,18].

In this context, the present paper describes the possibility of employing tannic acid for the obtainment of natural coatings on two different magnesium alloys, AZ31 (wt%: Mg 95.9, Al 3.1, Zn 0.73, Mn 0.25, Si 0.02) and AZ91 (wt%: Mg 90.21, Al 8.8, Zn 0.68, Mn 0.30, Si 0.01) [19], widely investigated for biomedical applications [2] and intended for cardiovascular and orthopedic applications, respectively, in order to control their degradation rate. The coating developed for AZ91 by Spriano et al. [20] was employed on AZ31 too. Due to the poor performance of such surface modification, a new protocol was tested for the protection of AZ31. Both the tannic acid coatings on AZ31 and AZ91 were deeply characterized.

## 2. Materials and Methods

### 2.1. Samples Preparation

AZ91 plane samples (11 × 11 × 2 mm) were prepared by the combination of 3D printing and investment casting, as described in [20]. AZ91 samples were washed for 5 min in ethanol and 5 min in ultrapure water in an ultrasonic bath, dried, and then coated with tannic acid by immersion in a 5 mg/mL aqueous solution of tannic acid (TA, tannic acid 403040-100G Sigma Aldrich, St. Louis, MO, USA) for 3 h at 37 °C, as described in [20]. Coated samples will be indicated as AZ91TA_5 from now on.

AZ31 plane samples (11 × 11 × 1 mm) were cut from AZ31 plates (GoodFellow Cambridge Limited—UK, Huntingdon, UK), washed for 5 min in ethanol and 5 min in ultrapure water in an ultrasonic bath, dried, and then coated with tannic acid by immersion in a 5 mg/mL aqueous solution of tannic acid (TA, tannic acid 403040-100G Sigma Aldrich, St. Louis, MO, USA) for 3 h at 37 °C or by immersion in a 20 mg/mL aqueous solution of tannic acid for 30 min at 37 °C. Hereafter, the sample coated using the former protocol will be labeled as AZ31TA_5, while the ones that underwent the latter procedure will be referred to as AZ31TA_20.

### 2.2. Surface Characterization

Surface morphology and semi-quantitative chemical composition were investigated using scanning electron microscopy equipped with energy dispersive spectroscopy (SEM, JEOL, JCM 6000 plus, EDS, JEOL, and JED 2300, Tokyo, Japan).

The samples underwent morphological characterization using an optical profilometer (LSM900, ZEISS, Oberkochen, Germany), and the topographical parameters were assessed following ISO 25178 [21]. The software ConfoMap® ST (Version 7.4.8341, ZEISS, Oberkochen, Germany) was utilized to analyze the height maps derived from the confocal microscope. 

Attenuated total reflection Fourier transform infrared (ATR-FTIR) spectroscopy (FTIR Hyperion 2000–Tensor 27, Bruker Optics, Ettlingen, Germany) was performed to investigate the surface chemistry. Spectra were collected in the wavenumber range between 4000 and 600 cm^−1^, acquiring 64 scans for each spectrum with a resolution of 5 cm^−1^. The background was collected before each measurement.

Surface composition and characteristic chemical functionalities were investigated through X-ray photoelectron spectroscopy (XPS, PHI 5000 Versaprobe II, ULVAC-PHI, Inc., Kanagawa, Japan). Survey spectra and high-resolution ones for C, O, and Mg were acquired. Charging effects were corrected by fixing the C1s peak binding energy (BE) at 284.8 eV [22] and the element peaks were fitted using Casa XPS software (Version 2.3.25PR1.0, Casa Software Ltd., Teingmouth, UK) [23] using a Shirley baseline and a Gaussian–Lorentzian peak shape [24].

### 2.3. Degradation and Corrosion Studies 

Degradation tests were performed in PBS (phosphate-buffered saline, 79382, 50 tabs, Sigma Aldrich, St. Louis, MO, USA) for 1, 2, 7, and 14 days following ASTM G31-72 standard [25]. After different soaking times samples were washed, dried, and observed at SEM. pH and magnesium concentrations were analyzed in soaking solutions at each time. Mg concentration in the PBS solution was measured by a photometric method (Hanna Instruments, Woonsocket, RI, USA).

Corrosion studies were performed at the room temperature of about 23–25 °C in the electrochemical environment using phosphate-buffered saline (PBS), Hanks’ balanced salt solution with Ca^2+^ and Mg^2+^ ions (HBSS^++^, Lonza, Vivers, Belgium), Hanks’ balanced salt solution without Ca^2+^ and Mg^2+^ ions (HBSS^−^, Lonza, Vivers, Belgium), Gibco^TM^ Dulbecco’s Modified Eagle Medium (DMEM, Thermo Fisher Scientific, Waltham, MA, USA), 0.9% NaCl, and Ringer solution (B. Braun, Hessen, Germany) to study the effect of different ions on the corrosion behavior of the tannic acid coating.

#### 2.3.1. Electrochemical Cell

The electrochemical cell with standard three-electrode system was used. AZ31 and AZ91 surfaces were placed as the working electrode (WE). The reference electrode (RE) used for this study was a saturated calomel electrode (Hg_2_Cl_2_ (SCE)) KE10 (Sensortechnik Meinsberg, Waldheim, Germany), whereas a platinum rod (3 mm diameter) was used as a counter electrode (CE). The potentiostat Autolab PGSTAT204 (Metrohm, Herisau, Switzerland) was used to conduct the potentiodynamic measurements. The potentiostat programming and data recording were performed by Nova advanced electrochemical software 2.x (Metrohm, Herisau, Switzerland).

#### 2.3.2. Electrochemical Corrosion Tests

Potential stabilization was first achieved as mentioned in [26] before measuring open-circuit potential (OCP). An amount of 50 mL of test fluid was used for each sample with an exposed surface area of 28.27 mm^2^. Following the stable OCP, current density (I (mA/cm^2^)) was recorded as a function of the WE potential (E (V) vs. SCE). At least five potentiodynamic measurements were performed for each sample in each test fluid. For every sample, the potentiodynamic polarization was varied from −2.0 to −1.0 V at a scan rate of 10 mV/s. An amount of 1 mV was chosen as the suitable step size between two points. The logarithmic values of the current density were plotted against the WE potential to obtain the Tafel curve. Similarly, these obtained Tafel curves were extrapolated using Nova advanced electrochemical software 2.x (Metrohm, Herisau, Switzerland) to obtain the corrosion current density (icorr) and the corrosion potential (Ecorr). These Tafel curves were reproduced using Origin 2022 (Origin Lab Corporation, Northampton, MA, USA). The following equation in accordance with ASTM G102-89 standard [27] was used to calculate the corrosion rates:

(1) $$CR=K_{1}\frac{i_{corr}}{\rho }EW$$

where *CR* is the corrosion rate (mm/yr), *K*_1_ is the constant and its value is 3.27 × 10^−3^ (mm g/μA), *i_corr_* is the corrosion current density (μA/cm^2^), *ρ* is the standard density (g/cm^3^), and *EW* is the equivalent weight (g/eq). The *EW* and ρ were 12.3 g/eq and 1.74 g/cm^3^ for AZ31 [19], respectively, and 11.89 g/eq and 1.81 g/cm^3^ for AZ91 [28], respectively.

## 3. Results and Discussion

**Figure 1:**
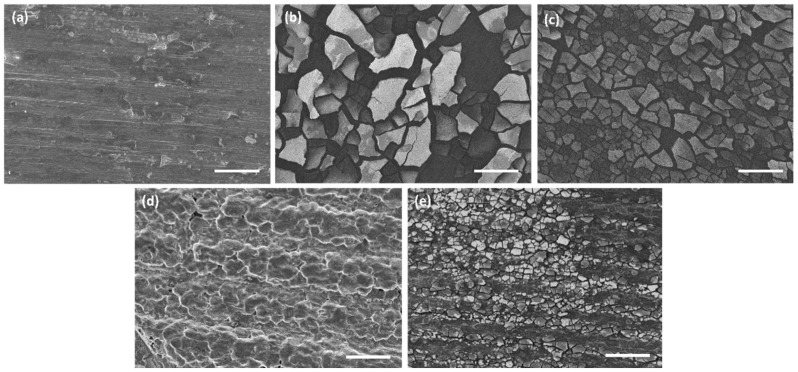
SEM images (100×) of (**a**) AZ91 uncoated, (**b**) AZ91TA_5, (**c**) AZ31 uncoated, (**d**) AZ31TA_5, (**e**) AZ31TA_20. Markers are 200 µm each.

Figure 1 reports SEM images of Mg alloys before and after tannic acid coating.

The AZ91 bare sample (Figure 1a) presents a rough surface that resembles the layered topology of the 3D-printed polymer pattern replicated by investment casting (Figure S1 in Supplementary Material ). After tannic acid coating (Figure 1b) the main topography is maintained but plenty of micro-scales completely cover the surface, confirming surface reaction and coating, as reported in [20].

The surface of the bare AZ31 sample (Figure 1c) appears as a typical rolled metal with some scratches and scales, attributable to surface deformation and oxidation during working. After tannic acid coating in a 5 mg/mL solution for 3 h (Figure 1d), analogously to AZ91 substrates, a lot of big (hundreds of microns) scales cover the surface. When coating is performed in a 20 mg/mL TA solution for 30 min (Figure 1e), scales have smaller dimensions (not so far from the ones obtained on AZ91 samples) and cover the surface in a more homogeneous way (less detachment). The presence of the phenolic coating is also confirmed by EDS analyses (Table S1 in Supplementary Material), where an increase in the C content was detected after the treatments on both AZ31TA_5 and AZ3TA_20. No differences in the two coatings were observed in terms of the chemical composition.

Since roughness is a fundamental aspect concerning biomaterial implants for both bone contact and cardiovascular applications [29], profilometry was used to evaluate how the TA coating can change the alloy’s topography. It is to be noticed that it is not within the intent of this paper to obtain specific values of surface roughness: the aim is limited to evaluating the changes in the surface morphology upon coating with tannic acid. According to SEM observation (Figure 1), the deposition of a TA coating on both AZ31 and AZ91 provokes changes in the surface roughness, S_a_ (Table 1). The formation of scales and cracks on the coated AZ31 results in increased S_a_ values: the rougher surfaces were the ones treated for 3 h with a 5 mg/mL TA solution, while a minor increment was observed for the samples soaked for only 30 min in a 20 mg/mL TA medium. As expected, by lowering the treatment time, the reactions that occur on the surface of AZ31 produce a thinner reaction layer, which is less subjected to cracking upon drying. On the contrary, the AZ91 roughness was lowered by the surface coating. Thanks to the higher corrosion resistance of this alloy compared to the AZ31, the formed layer is more adherent to the surface, with fewer cracks and smaller scales, and it evens the irregularities of the samples.

**Figure 2:**
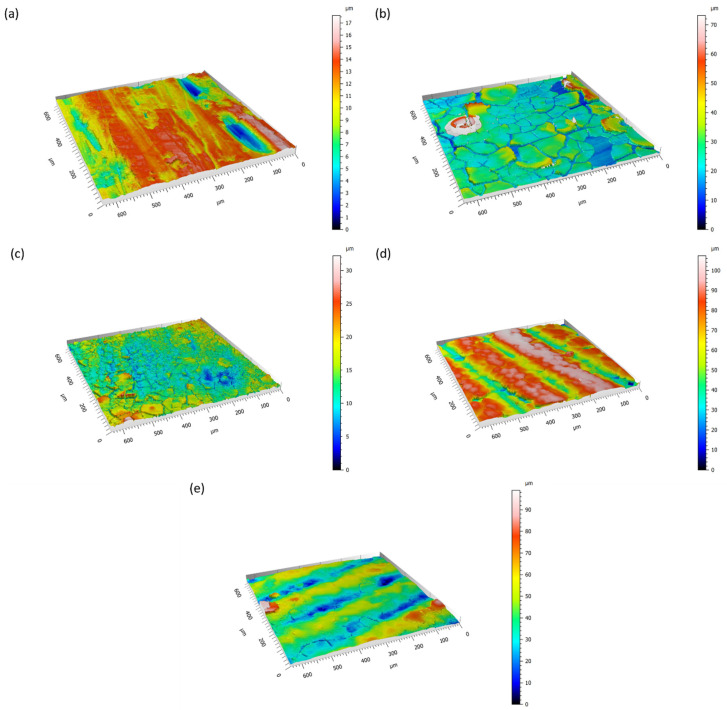
Three-dimensional reconstruction of (**a**) AZ31, (**b**) AZ31TA_5, (**c**) AZ31TA_20, (**d**) AZ91, (**e**) AZ91TA obtained by optical profilometry.

The formation of scales of different dimensions on AZ31 samples and the flattening effect of the TA coating of AZ91 are well visible in the 3D reconstruction of the surfaces obtained by optical profilometry (Figure 2).

The ability of the coating to reduce and slow the corrosion of the magnesium AZ31 alloy was evaluated by soaking the samples with different treatments in PBS. The formation of degradation products, pH increases, and Mg release in the solution were studied as main indicators of surface corrosion processes.

**Figure 3:**
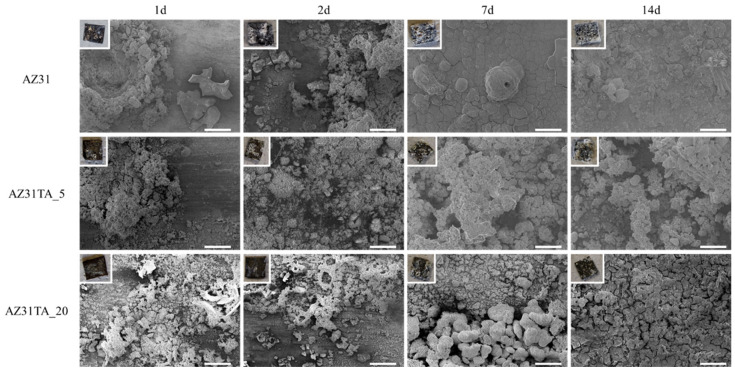
SEM images of AZ31, AZ31TA_5, and AZ31TA_20 samples after degradation in PBS at different time points, namely 1, 2, 7, and 14 days. Markers are 200 µm each. Optical pictures of the samples are shown in the insets of the images.

As possible to see by SEM micrographs (Figure 3), degradation products are promptly formed on the surface of both uncoated and coated AZ31 samples, as early as after 1 day of soaking in PBS. EDS chemical analysis (Table S2 in Supplementary Material) showed that the corroded layer is mainly formed by magnesium oxides and phosphate species [30]. The degradation products grew constantly with increased soaking time, covering all the surfaces with white oxide flakes, and were observed at a macroscopic scale (Figure 3 insets).

From the optical and SEM observation of the samples, it seems that a thicker and more compact oxide layer is formed on the surface of the bare AZ31 alloy, compared to the coated surfaces, and that the coating on AZ31TA_20 is more effective than the one on AZ31TA_5 to slower the corrosion of magnesium.

**Figure 4:**
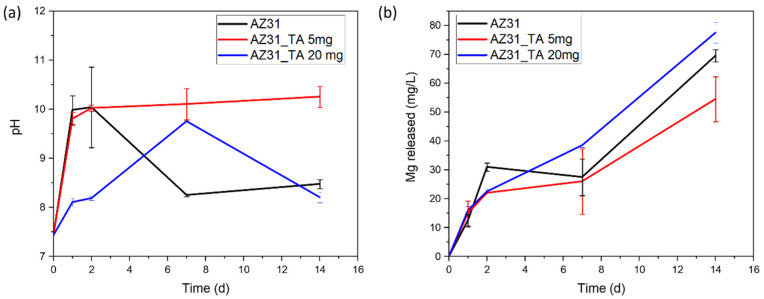
Degradation test in PBS, (**a**) pH trend versus soaking time, (**b**) Mg release vs. soaking time.

Figure 4 reports pH and magnesium ions release in PBS during sample soaking for up to 14 days. A rapid pH increase, up to 10, can be observed after 1 d for both AZ31 and AZ31TA_5, while AZ31TA_20 can maintain solution pH close to 8 up to 2 d. A pH decrease of around 8 after 7 or 14 days can be recorded for AZ31 and AZ31TA_20, respectively, while AZ3TA_5 induces persistent basification up to 14 d. The differences, mainly visible at 7 days of soaking, can be correlated with the dissolution and deposition of degradation products. No significant differences can be denoted among samples considering Mg release.

Looking at these results, AZ31TA_20 seems more promising than AZ31TA_5. Degradation behavior is in accordance with SEM observations; in fact, AZ31_TA 20 mg presents smaller scales and cracks, suggesting better coverage and protection. However, it seems that the ability of the tannic acid coating to protect Mg substrates from degradation in PBS is higher on AZ91 substrates [20] than on AZ31. This behavior can be attributed both to a lower reactivity of AZ91 substrates, as reported in [19], and to a better coverage (fewer cracks) of the obtained TA coating.

According to these results, the coating obtained using a TA concentration of 5 mg/mL in the processing solution was not effective in preventing the corrosion of the AZ31 substate, therefore it was not investigated further. Deeper studies on the surface chemistry of AZ31TA_20 and AZ91TA were performed by FTIR and XPS.

**Figure 5:**
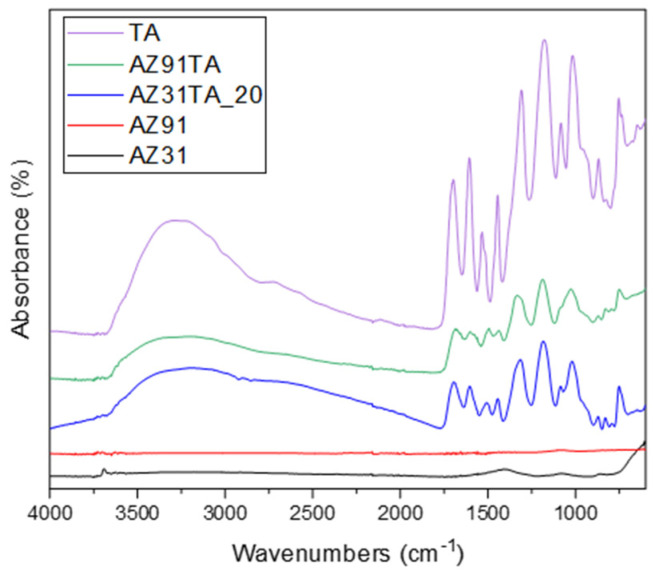
FTIR spectra of bare AZ31 and AZ91, of Mg samples after coating with tannic acid (AZ31_TA 20 mg/mL and AZ91_TA), and of tannic acid (TA).

The FTIR spectra are reported in Figure 5. As expected, the untreated metal surfaces have almost no absorption in the IR region, but a weak band around 1400 cm^−1^ was detected on AZ31, which can be related to some carbonates on the surface of the alloy [31]. After the coating formation, the typical fingerprint of tannic acid can be observed in the spectra, with the bands rising from the vibrational mode of phenolic OH (the halo at 3300 cm^−1^), C=O (at 1700 cm^−1^), aromatic rings (1700–1400 cm^−1^), COOH (1400–1200 cm^−1^), and C-O (1200–1000 cm^−1^) [32,33,34]. The higher intensity of the organic peaks on the AZ31TA_20 with respect to the AZ91TA is related to more intimate contact between the sample and the ATR crystal, due to the lower roughness of the AZ31 samples, which results in better signal detection. FTIR spectra are a further confirmation of the successful integration of tannic acid within the coating on the different alloys.

The surface chemistry of as received and coated Mg alloys was investigated by XPS survey spectra and reported in Table 2. The main elements of the alloys, namely Mg and Al, were detected even with different compositions than the nominal ones [19]. This is due to the presence of a native oxide layer, mainly composed of Mg and O, and high levels of C-containing and other kinds of contaminations. 

After the coating process, a decrease in the Mg and O contents and an increase in C on both types of samples were observed. These changes are much more intense on AZ91TA than on AZ31TA_20, as also possible to see by the C/Mg ratio (Table 2). On the former surface, the Mg is very low, and the C/Mg ratio has almost a tenfold increase, while on AZ31TA_20 Mg is about 8.6% and the C/Mg ratio is increased roughly two and half times. These differences can be related to the higher integrity of the TA coating on AZ91 samples, with respect to the AZ31 alloy. The cracks on the AZ31TA_20 coating expose the underneath metal surface, therefore increasing the amount of Mg detected.

**Figure 6:**
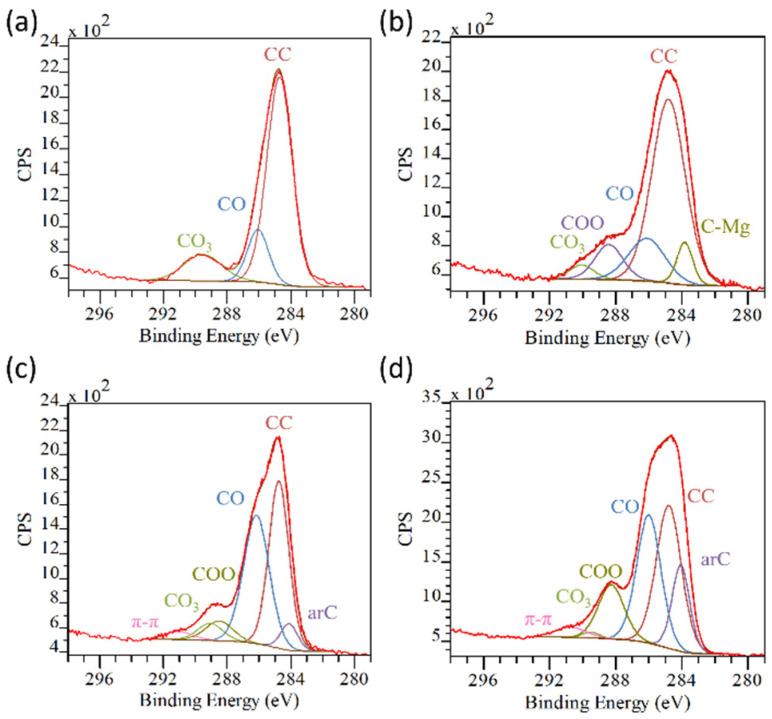
C1s peak deconvolution of: (**a**) AZ31; (**b**) AZ91; (**c**) AZ31TA_20; and (**d**) AZ91TA. In each image: red line: experimental spectrum; brown line: fitted spectrum; light brown line: baseline.

Further information on the surface chemistry can be drawn by analyzing the high-resolution peaks. Before coating, the C1s of both AZ31 and AZ91 (Figure 6a,b) are composed of signals arising mainly from carbonates (CO_3_, BE ≈ 289–290 eV) and carbonaceous contaminations (CO, BE ≈ 286 eV; COO, BE ≈ 288 eV) [24]. A contribution by C-Mg bonds (BE ≈ 284 eV) [35] is observed in the AZ91 pristine sample, probably due to some reaction occurring when the molten metal is cast into a ceramic mold with residues after burning a 3D polymeric pattern. The deposition of a TA-containing coating, following the surface modification, can be observed by the increase in the CO and COO components of the C1s peak (Figure 6c,d; Table S3 in Supplementary Material) and the appearance of the peaks related to aromatic C (arC, BE ≈ 284 eV) [36] and the delocalized electron of the π–π aromatic bonds (BE ≈ 291 eV) [37]. These chemical groups and bonds are very typical of the TA, as possible to see by its chemical formula (Figure S2 in Supplementary Material). The lower intensity of the TA peaks on coated AZ31 with respect to the coated AZ91 can be related to the presence of larger cracks in the coatings on the former surface, which therefore exposes uncoated parts, in agreement with the compositional results (Table 2).

**Figure 7:**
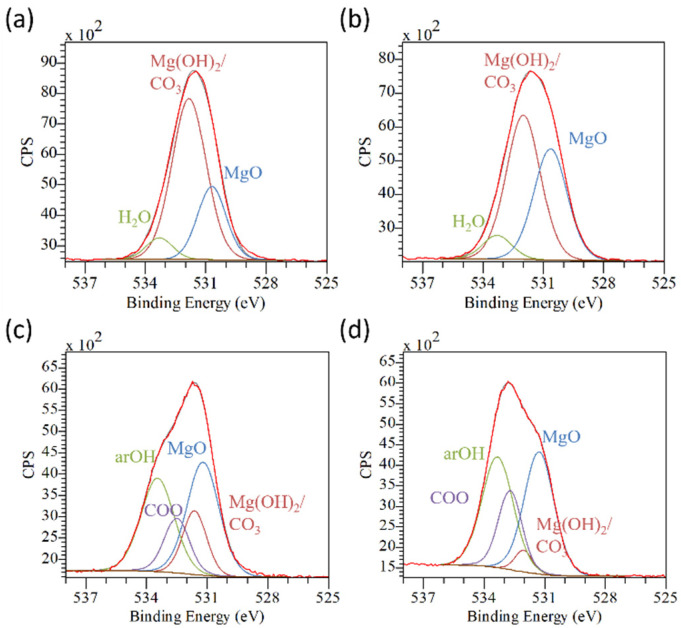
O1s peak deconvolution of: (**a**) AZ31; (**b**) AZ91; (**c**) AZ31TA_20; and (**d**) AZ91TA. In each image: red line: experimental spectrum; brown line: fitted spectrum; light brown line: baseline.

Similar results were found observing the O1s peaks. The pristine alloys (Figure 7a,b) show the expected components of the magnesium native oxide layer, a mixture of oxide MgO (BE ≈ 530.5 eV) and hydroxide Mg(OH)_2_ (BE ≈ 531.6) [13,24,38]. Adsorbed water was also found (BE ≈ 533.5) [24]. On the other hand, the coated surfaces have very strong contributions by the aromatic hydroxyl groups (BE ≈ 533.5) and a smaller one by COO groups (BE ≈ 531.5) [36] of the tannic acid. Again, the higher contribution of the organic molecules in the coating was observed for the AZ91TA surface, sustaining the hypothesis of a more continuous layer. The fact that the amount of magnesium hydroxide is much higher than the one of magnesium oxide in the coating (Table S4 in Supplementary Material) suggests that a reaction occurs during the soaking in the TA solution, with the formation of mainly MgO instead of Mg(OH)_2_ and the formation of a mixed layer TA-Mg oxide.

**Figure 8:**
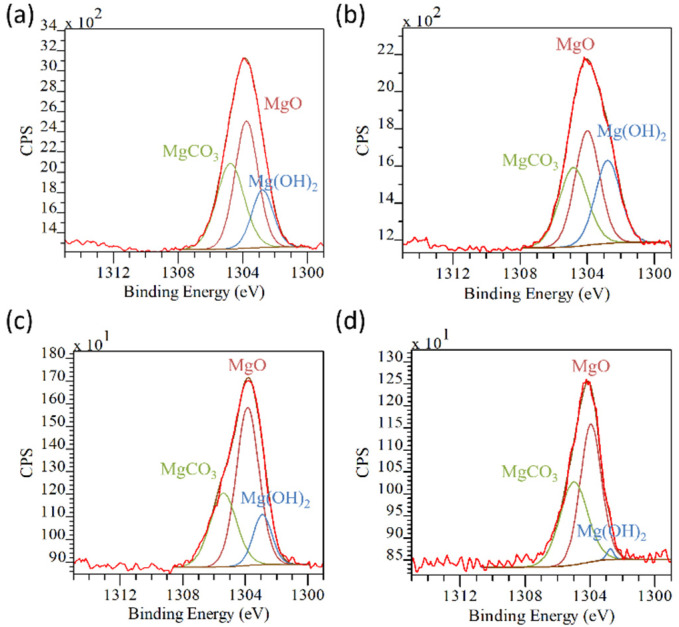
Mg1s peak deconvolution of: (**a**) AZ31; (**b**) AZ91; (**c**) AZ31TA_20; and (**d**) AZ91TA. In each image: red line: experimental spectrum; brown line: fitted spectrum; light brown line: baseline.

The hypothesis on the evolution of the oxide layer based on the oxygen spectra is confirmed by the deconvolution of the Mg1s peaks. Concerning the untreated alloys, the surface is composed of a mixture of mainly magnesium oxide (BE ≈ 1303.7), along with hydroxide (BE ≈ 1302.5) and carbonates (BE ≈ 1304.6) (Figure 8a,b; Table S5 in Supplementary Material) [15,18]. When the coating is formed on the alloys, a reduction of the components related to the magnesium hydroxide and carbonates was observed, while the MgO increased on both treated AZ31 and AZ91 (Figure 8c,d; Table S5 in Supplementary Material). These results strengthened the suggestion of the formation of MgO instead of Mg(OH)_2_ during the reaction with the TA solution. 

**Figure 9:**
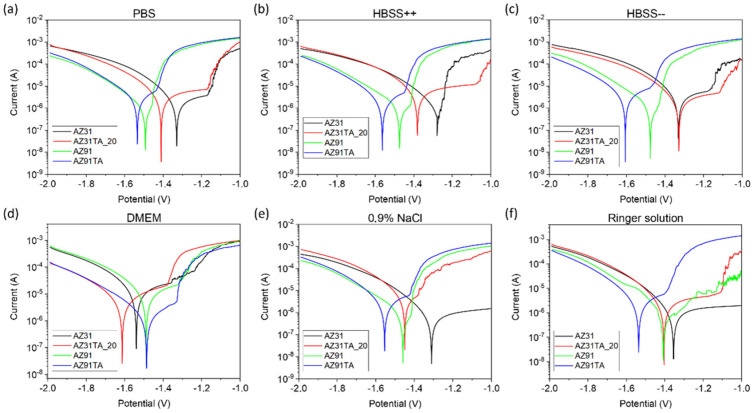
Tafel plots of all samples in the different test fluids: (**a**) PBS; (**b**) HBSS, (**c**) HBSS w/o Ca Mg; (**d**) DMEM; (**e**) 0.9% NaCl; and (**f**) Ringer solutions.

Figure 9 shows the Tafel plots obtained by potentiodynamic polarization measurements. The shifting of corrosion current potential was observed to a more negative side after coating for both AZ31 and AZ91 in the PBS, HBSS, 0.9% NaCl, and Ringer solutions. This indicates the weakening of the barrier for corrosion [25,39]. However, no such shift was observed for AZ91TA in DMEM. Similarly, AZ31TA_20 also showed no shifting in corrosion potential (Ecorr) in HBSS (without Ca^2+^ and Mg^2+^ ions). This randomness in the shifting of the Ecorr might be because of the random scales and cracks present on the coated samples and the site of the sample surface exposed to the fluid and it is difficult to predict the real mechanism behind it. Coatings with fewer or no cracks can reduce this uncertainty.

**Figure 10:**
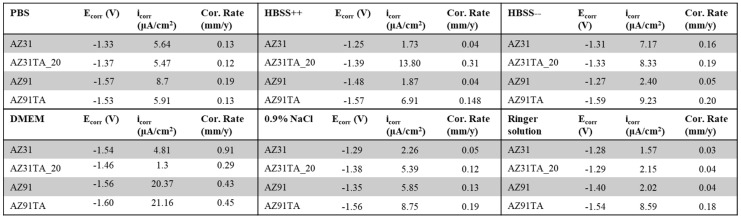
Corrosion parameters of all samples in PBS; HBSS++; HBSS--; DMEM; 0.9% NaCl; and Ringer solution.

Figure 10 shows the values of the E_corr_, i_corr_, and corrosion rates for all the samples. The E_corr_ values for AZ31, AZ31TA, AZ91, and AZ91TA were in the range of ((−1.25)−(−1.54) V), ((−1.28)−(−1.46) V), ((−1.27)−(−1.57) V), and ((−1.53)−(−1.61) V), respectively, in all fluids. The highest values of Ecorr were observed in DMEM for AZ31, AZ31TA, and AZ91TA, whereas AZ91 showed the highest value for corrosion potential in the PBS solution. However, there was no influence of ions that could be observed on the corrosion potential due to the randomness of values in different fluids. Similar kinds of variations in i_corr_ and corrosion rates were also observed. For instance, the measured i_corr_ was more than 20 µA/cm^2^ in DMEM for AZ91 and AZ91TA. However, it was even less than 10 µA/cm^2^ in other fluids for the same samples. Moreover, AZ31 showed the highest corrosion rate of 0.91 mm/y in the DMEM as well. Interestingly, corrosion rates were increased in coated samples (AZ31TA, AZ91TA) as compared to the uncoated samples (AZ31, AZ91) for most of the fluids, exceptions are DMEM and PBS. Due to the huge variation in crack dimensions, we can assume that the surface of the same sample was not even similar enough to establish the direct relationship of the ions on the variation of the corrosion parameters. It can be reported that the corrosion rates were increased due to the presence of micro-cracks in the coating, as reported in [40]. Obtaining a crack-free coating or a coating with minimal cracks is already a challenging task in the case of natural molecules [20]. We aim to further optimize the coating parameters in future studies to obtain the best possible coating to have good corrosion resistance as reported in [40].

## 4. Conclusions

The control of the degradation rate is still a major issue for the clinical application of Mg alloy-based devices. In this paper, a tannic acid coating was proposed for the AZ31 alloy with a double scope: slow the corrosion of the implant and exploit the beneficial properties of polyphenols. The same approach was effective in reducing the static degradation of AZ91, as proven by the authors in a previous study [20].

Here, the TA coating was unable to protect the AZ31 substrate from static degradation, due to the elevated reactivity of this Mg alloy that produced a highly cracked and poorly attached TA-Mg oxide coating, despite the use of different coating protocols. Furthermore, the electrochemical tests showed that both for AZ31 and AZ91, the coating worsened the corrosion behavior by increasing the corrosion current and the corrosion rate, as consequences of the formation of galvanic cells between the coating scales and the exposed metallic substrate. However, these results are valuable since they provide important information towards the development of protective coatings for Mg alloys, by emphasizing the uttermost importance of obtaining homogeneous and crack-free layers. In fact, current studies in our group are devoted to solving this issue and they will be the object of future publications.

## Figures and Tables

**Table 1:** Roughness, S_a_, of the different samples obtained by ISO 25178.

	AZ31	AZ31TA_5	AZ31TA_20	AZ91	AZ91TA
S_a_ (µm)	1.57	5.66	3.72	14.2	10.3

**Table 2:** Elemental composition (at%) of the main elements in the different samples and their ratios.

	Element (at%)						Ratio
	Mg	O	C	Al	Zn	Others	C/Mg
AZ31	14.2	43.2	25.4	1.1	-	16.1	1.8
AZ31TA_20	8.6	38.6	43.4	1.6	0.1	7.7	5.0
AZ91	10.6	39.7	29.6	3.2	-	16.9	2.7
AZ91TA	2.9	33.7	61.4	-	-	2	21.2

## Data Availability

Data are available upon request to the authors.

## Supplementary Materials

The following supporting information can be downloaded at: https://www.mdpi.com/article/10.3390/ma17020343/s1, Figure S1: Optical picture of the AZ91 sample surface, Figure S2: Tannic acid formula, Table S1: Surface composition by EDS of AZ31 samples as received and after coating with TA solution at different concentrations, Table S2: Surface composition by EDS of AZ31, AZ31TA_5, and AZ31TA_20 after soaking in PBS solution for different time points, Table S3: Composition of the C1s peak (at%), Table S4: Composition of the O1s peak (at%), Table S5: Composition of the Mg1s peak (at%).
