# Supplementary material for: Characterization of Tannic Acid-Coated AZ31 Mg Alloy for Biomedical Application and Comparison with AZ91
Source: Materials (Basel). 2024 Jan 10;17(2):343. doi: 10.3390/ma17020343 (PMC10817444; doi:10.3390/ma17020343)
Supplement: Supplementary file 1 [file materials-17-00343-s001.zip › materials-2799674-supplementary.pdf]

materials

Supporting information

Characterization of tannic acid coated AZ31 and AZ91 Mg alloys for biomedical applications

Jacopo Barberi[1]*, Muhammad Saqib[2], Anna Dmitruk[3], Jörg Opitz[2], Krzysztof Naplocha[3], Natalia Beshchasna[2], SilviaSpriano[1], Sara Ferraris[1]*

[1] Politecnico di Torino, Department of Applied Science and Technology, Turin, Italy; jacopo.barberi@polito.it, silvia.spriano@polito.it, sara.ferraris@polito.it

[2] Fraunhofer Institute for Ceramic Technologies and Systems IKTS, Dresden, Germany; muhammad.saqib@ikts.fraunhofer.de, joerg.opitz@ikts.fraunhofer.de, natalia.beshchasna@ikts.fraunhofer.de

[3] Department of Lightweight Elements Engineering, Foundry and Automation, Faculty of Mechanical Engineering, Wroclaw University of Science and Technology, Wroclaw, Poland; anna.dmitruk@pwr.edu.pl, krzysztof.naplocha@pwr.edu.pl

* Corresponding author: jacopo.barberi@polito.it, sara.ferraris@polito.it; Tel.: +3901109043231

AZ91 samples

The morphology at the macroscale of the casted samples of AZ91 is shown in Figure S1.

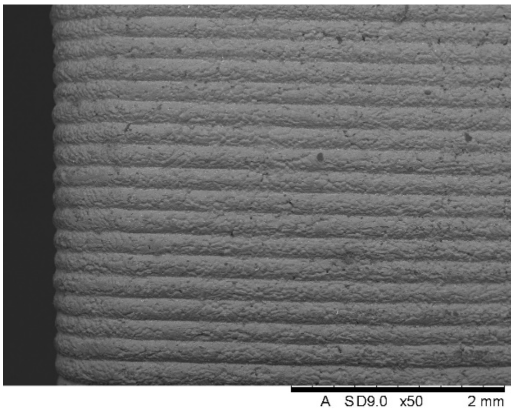

Figure S1: Optical picture of the AZ91 sample surface.

AZ31 tannic acid coating characterization

The results obtained by EDS on AZ31 samples before and after coating with the two different TA solutions are reported in **Table S1**.

Table S1: Surface composition by EDS of AZ31 samples as received and after coating with TA solution at different concentrations.

	Elemental composition (at%)			
	C	O	Mg	Al
**AZ31**	7.8	36.1	54.5	1.6
**AZ31TA_5**	37.3	48.2	14	0.5
**AZ31TA_20**	35.6	47.1	16.7	0.6

Degradation studies

The chemical composition of the different AZ31 samples after degradation up to 14 d in PBS, evaluated by EDS, are reported in Table S2.

Table S2: Surface composition by EDS of AZ31, AZ31TA_5, and AZ31TA_20 after soaking in PBS solution for different time points.

	Time (day)	Elemental composition (at%)					
		C	O	Na	Mg	Al	P
**AZ31**	1	4.9	54.9	1.6	33.2	1.4	4.0
	2	12.3	52.2	2.4	28.0	1.4	3.0
	7	1.0	67.6	2.1	20.4	0.2	8.6
	14	7.5	65.7	3.4	12.6	0.5	10.0
**AZ31TA_5**	1	7.7	52.0	1.3	34.4	N.D.	4.6
	2	6.1	63.3	2.3	21.9	0.4	5.9
	7	2.5	70.5	3.4	14.0	0.0	9.2
	14	N.D.	77.9	0.5	13.6	0.3	7.7
**AZ31TA_20**	1	3.5	61.2	2.7	23.6	1.1	7.9
	2	11.1	59.9	2.2	19.6	0.5	6.8
	7	4.2	68.7	2.4	15.5	0.7	8.4
	14	12.3	64.2	2.8	12.4	0.7	7.7

XPS peak deconvolution

The composition of the C1s, O1s and Mg1s peaks obtained by deconvolution are reported in Table S3, Table S4, and Table S5, respectively.

Table S3: Composition of the C1s peak (at%).

**C1s**	**CC**	**CO_3**	**CO**	**arC**	**COO**	**π-π/C-Mg**
**AZ31**	71.48	13.57	14.94			
**AZ31TA**	40.63	5.17	39.63	5.38	6.45	2.74
**AZ91**	62.84	3.60	16.17		10.35	7.03
**AZ91TA**	35.89	0.92	31.05	15.19	13.89	3.07

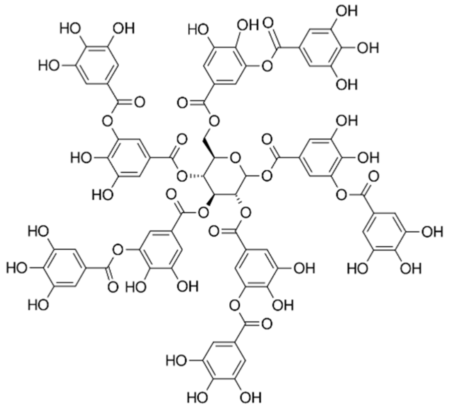

Figure S2: Tannic acid formula.

Table S4: Composition of the O1s peak (at%).

O1s	$Mg(OH)_2/CO_3$	H_2O	arOH	MgO	COO
**AZ31**	67.4	7.0		25.6	
**AZ31TA**	15.8		32.0	35.5	14.7
**AZ91**	52.9	7.3		39.8	
**AZ91TA**	4.1		36.8	38.8	22.3

Table S5: Composition of the Mg1s peak (at%).

Mg1s	MgO	$MgCO_3$	$Mg(OH)_2$
**AZ31**	44.90	36.14	18.96
**AZ31TA**	55.55	30.06	14.39
**AZ91**	39.50	31.50	29.01
**AZ91TA**	55.22	43.34	1.44
